# Whole-genome sequencing of a laboratory-evolved yeast strain

**DOI:** 10.1186/1471-2164-11-88

**Published:** 2010-02-03

**Authors:** Carlos L Araya, Celia Payen, Maitreya J Dunham, Stanley Fields

**Affiliations:** 1 Department of Genome Sciences, University of Washington, Box 355065, Seattle, Washington, 98195 USA; 2 Department of Medicine, University of Washington, Box 355065, Seattle, Washington, 98195 USA; 3 Howard Hughes Medical Institute, University of Washington, Box 355065, Seattle, Washington, 98195 USA

## Abstract

**Background:**

Experimental evolution of microbial populations provides a unique opportunity to study evolutionary adaptation in response to controlled selective pressures. However, until recently it has been difficult to identify the precise genetic changes underlying adaptation at a genome-wide scale. New DNA sequencing technologies now allow the genome of parental and evolved strains of microorganisms to be rapidly determined.

**Results:**

We sequenced >93.5% of the genome of a laboratory-evolved strain of the yeast *Saccharomyces cerevisiae *and its ancestor at >28× depth. Both single nucleotide polymorphisms and copy number amplifications were found, with specific gains over array-based methodologies previously used to analyze these genomes. Applying a segmentation algorithm to quantify structural changes, we determined the approximate genomic boundaries of a 5× gene amplification. These boundaries guided the recovery of breakpoint sequences, which provide insights into the nature of a complex genomic rearrangement.

**Conclusions:**

This study suggests that whole-genome sequencing can provide a rapid approach to uncover the genetic basis of evolutionary adaptations, with further applications in the study of laboratory selections and mutagenesis screens. In addition, we show how single-end, short read sequencing data can provide detailed information about structural rearrangements, and generate predictions about the genomic features and processes that underlie genome plasticity.

## Background

Despite the wealth of knowledge that comparative genomics has provided about the evolution of past and current life forms, the process of adaptation is still poorly understood. Predictive insight into how adaptation will occur -- which adaptive mutations are likely to arise and fix during selection -- is a daunting challenge. Progress in this area will further efforts to deter cancer progression, the emergence of new pathogens, and antibiotic resistance. As a simple model for analyzing adaptation, experimental evolution of microorganisms provides the unique opportunity to catalog, monitor the dynamics, and measure the reproducibility of adaptation in real time. In the past decade, the characterization of laboratory evolution outcomes beyond phenotypic analysis has been advanced by the use of DNA microarrays. In bacteria and yeast, these approaches have been applied to expose adaptive point mutations and structural rearrangements in response to carbon source replacement as well as carbon source and nutrient limitation [1-4]. Furthermore, studies have begun to unveil the effect of population dynamics on the outcomes of evolution, providing direct evidence of parallel evolution and clonal interference and support for historical contingencies [4-7]. However, these studies are limited in that the precise nature of the adaptive point mutations or large-scale sequence rearrangements in the evolved organisms has not been defined systematically.

In recent years, however, a series of high-throughput DNA sequencing technologies capable of producing gigabases of DNA sequence information in a single experiment have been developed. These technologies are transforming biological research, allowing the rapid identification of genetic variation intrinsic to diseases, behavior and other traits. Indeed, these technologies have been applied to a wide host of biological research problems, from enhancing our understanding of laboratory strains of bacteria by pinpointing suppressor mutations [8] and mutational biases [9], to identifying the genetic variation within all known serotypes of a major viral pathogen [10]. In yeasts, they have been applied to analyze the genetic variation within and between wild and domestic populations [11], the spectrum of spontaneous mutations in *Saccharomyces cerevisiae *[12] and the adaptive mutations in a *Pichia stipilis *strain efficient in xylose fermentation [13]. In bacteria, these sequencing technologies have recently been applied to study the rate of evolutionary adaptation over 40,000 generations [14]. Furthermore, they have launched a personal genomics era that is revealing the breadth of genetic variation in the small number of individuals fully sequenced thus far [15-18], and across targeted subgenomic portions in many individuals [19,20], pinpointing disease causative alleles in exomes of affected individuals [21,22].

Here, we use whole-genome sequencing to reveal the repertoire of point mutations and copy number polymorphisms in an evolved *S. cerevisiae *strain. We sequenced an adapted strain isolated after ~188 generations of a continuous haploid culture under sulfate limitation, and compared this analysis to mutational profiling data obtained using array-based technologies [4]. We generated high-depth sequencing data for the evolved and parental genomes, applied a heuristic approach to uncover single nucleotide polymorphisms (SNPs) and a recursive segmentation algorithm to discover breakpoints and refine copy-number estimates, and devised a general approach to extract precise breakpoint sequence information from single-end short reads. These breakpoints allowed us to delineate the structural rearrangement underlying a 5× copy number amplification of a ~11 kb genomic segment.

## Results

We constructed DNA sequencing libraries from genomic DNA of the yeast strain DBY11331, evolved in a sulfate-limited chemostat, and its haploid ancestor strain DBY10147 [4]. We collected 13,555,852 and 13,901,121 single-end, 36 bp reads from the evolved and parent genomes, respectively, on an Illumina Genome Analyzer II platform (using two lanes per strain). More than 75% of the reads (12,274,183 for the evolved genome; 10,441,548 for the parental genome) were aligned to the *S. cerevisiae *reference genome using *Maq *[23]. Following quality-filtering, these reads yielded a coverage of ~99.8% of the mappable nuclear genome of both strains with an average read-depth of 35.0× and 28.2× in the evolved and ancestor genomes, respectively [Additional File 1: Supplementary Table S1]. The majority of reads (>80%) had a mapping quality score ≥30.

### A heuristic approach to point mutation detection

In sequencing the adapted strain, our aim was to reveal the full complement of genetic adaptations that had occurred during its evolution under sulfate limitation. Copy number variation and point mutation analysis of this strain assessed by use of ORF array and genomic tiling array approaches, respectively, provided a data set against which to compare the efficacy of whole-genome sequencing [4]. To identify point mutations, we examined base calls using criteria that allowed comparisons between nucleotide positions in the evolved and parental short read data sets over the great majority of the genome. We performed SNP calling following two heuristic approaches, one that monitors commonly applied filters for read-depth and various quality scores, and one that is a simplified approach that monitors read-depth and the frequency of base-calls at each position. For the simplified approach, we required at least 6 reads per position with ≥80% concordant SNP calls in the evolved genome, and at least 5 reads per position with ≥70% concordant calls for a different base in the parental genome. This approach recapitulated known features in the evolved genome while maximizing breadth, yielding a high-confidence set of SNPs and that allowed us to examine up to 91% of the mappable bases in the yeast genome. We obtained modest gains in coverage following the simplified SNP calling approach [Additional File 1: Supplementary Tables S2 and S3].

**Figure 1:**
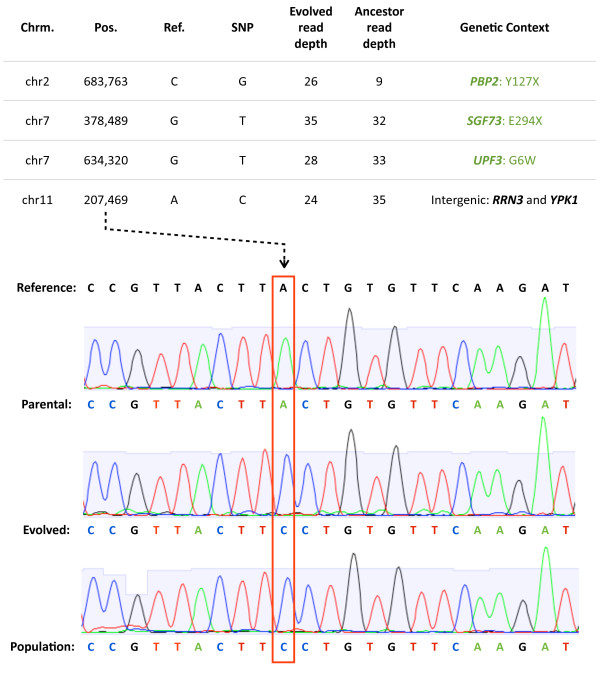
**Point mutation discovery in an evolved yeast genome**. SNPs that are supported by a read depth of ≥6 and ≥5 in the evolved and ancestor strain sequence data, respectively, are shown. These SNPS are also supported by ≥80% of the base calls for the position in the evolved strain and ≥70% of the calls in the parental strain. Tiling array-discovered SNPs in DBY11331 are labeled green. Sanger-sequence traces from evolved and ancestor genomes validate the SNP at chr11: 207,469 in the adaptive clone. Sanger sequence data derived from a population sample suggest that the chr11: 207,469 SNP is in high frequency in the evolving population time-point from which the evolved clone was isolated (bottom trace).

Applying these heuristics, we detected four single-point differences between the laboratory evolved strain and that of its parent: the previously-described point mutations at *PBP2 *(Y127X), *SGF73 *(E294X), and *UPF3 *(G6W) [4], and an additional point mutation intergenic to *RRN3 *and *YPK1 *(chr11: 207,469:A>C) which we confirmed by Sanger sequencing (Figure 1). The function of the RNA polymerase I-specific transcription initiation factor *RRN3 *is conserved between yeast and humans [24,25]. In humans, *TIF-IA *(the *RRN3 *ortholog) modulates ribosomal gene expression in response to nutrient limitation via the mTOR pathway [26]. The expression of *RRN3 *is less than half the level in the evolved strain compared to its ancestor, suggesting that this SNP may be regulatory and part of the adaptive response in this lineage. The finding of an additional SNP in the sequencing data but missed by array-based comparative genome sequencing is consistent with the estimated 15% false negative rate for the *SNPScanner *algorithm [3]. In addition, we searched for small insertions and deletions via gapped alignment of reads unmapped in the *Maq *alignment, requiring that candidate indel coordinates were sequenced as wildtype in the comparison strain. However, no small insertion or deletion differences between the evolved and parental genomes could be detected [Additional File 1: Supplementary Table S4].

### Segmentation-based detection of copy number polymorphisms

Previous analysis of the evolved and parental strains using comparative genome hybridizations on microarrays (array CGH) detected a genomic amplification spanning the high-affinity sulfate transporter *SUL1 *locus in the genome of the laboratory-evolved strain, providing a 50% fitness advantage relative to the ancestral genome [4]. *SUL1 *gene amplification was a common adaptation in continuous cultures under sulfate limitation, occurring in 15 of 16 adapted clones from various experimental evolutions [4]. However, the precise breakpoints and nature of the structural rearrangement has not been defined for these amplifications.

**Figure 2:**
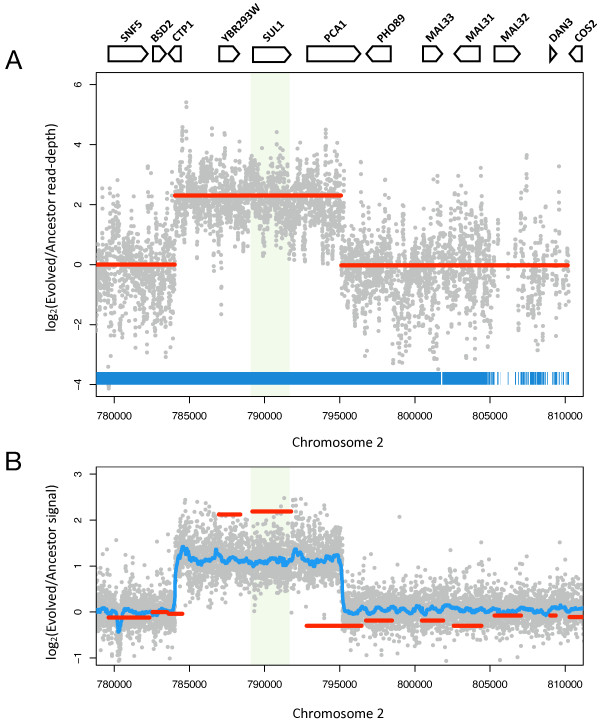
**A copy-number polymorphism harboring the *SUL1 *locus detected via whole genome sequencing**. A) Circular binary segmentation of sequencing-depth ratios between evolved and parental genomes using *DNAcopy*. Gray dots represent the per nucleotide read-depth in the genome sequencing data. Segmentation-derived regions of equal copy number are indicated in red, smoothed by removing segments < 3× standard deviation apart. Segmentation defines a ~11 kb region with a 5.0× amplification and predicted breakpoints at chr2:784,043-795,080 (+/- 25 bp). The region of the sulfate permease *SUL1 *gene is shaded green. Blue lines indicate mappable positions in the reference genome. B) Array CGH data are shown for comparison. Tiling array data (gray dots) support a copy number amplification with breakpoints at 784009-795143 (+/- 50 nt). Blue line corresponds to the R runmed-smoothed trend in the data. A copy number estimate of 4.5× was obtained by comparative hybridization using ORF arrays (red lines). Tiling array hybridizations were optimized for SNP-calling and as such provide inaccurate copy number estimates.

We compiled the read-depth data from evolved and parental genome sequencing data sets and calculated the ratio in read-depth for each base across the genome. We used these data to perform circular binary segmentation (CBS) with *DNAcopy*, a software package designed to partition the genome into segments of equal copy number from high-density array CGH data [27]. *DNAcopy *formulates the problem of copy-number variation (CNV) analysis as a change-point detection problem, whereby change-points correspond to the genomic locations of copy number transitions. The algorithm searches for change-points by recursively partitioning the genome and performing a maximal *t*-statistic test between segment ratios, joining segments with comparable ratios. We reasoned that this approach should detect transitions in ratios of sequencing read-depth as well, and applied it to search for such transitions throughout the yeast genome [Additional File 2: Supplementary Figure S1]. Indeed, segmentation of sequencing depth ratios detected an ~11 kb segment harboring *SUL1 *(chr2: 784,043-795,080 +/- 25 bp) with an estimated 5× amplification (Figure 2A). These breakpoint coordinates are in close proximity to estimated coordinates obtained from tiling array data (chr2: 784,009-795,143 +/- 50 bp) [4], but have narrower uncertainty windows (Figure 2B). The read depth-based copy number analysis yielded a value close to an integer, suggesting it may be a reliable approximation of genomic copy number.

### Breakpoint sequence determination and analysis of rearrangement structure

We sought to pinpoint with single-base resolution the structural rearrangements underlying the *SUL1 *amplification. To do so, we established a strategy to detect breakpoints in shotgun, single-end, short-read sequencing data. We unearthed breakpoint-spanning reads in the evolved genome sequencing data set by assembling unmapped reads into contigs using *Velvet*, a short-read *de novo *assembly algorithm [28]. These contigs were *BLAT*-aligned to the reference genome sequence in an attempt to detect signatures of novel structures in the genome. This approach yielded three contigs composed of subsequences with alignments to the mappable nuclear genome. Of these contigs, two aligned to sequences within the predicted amplification boundaries [Additional File 1: Supplementary Table S6]. Performing this analysis on the ancestor genome sequencing data yielded two contigs at distinct coordinates [Additional File 1: Supplementary Table S7], from which we estimate the likelihood of the signatures detected in the evolved genome arising independently of the amplification and found within the predicted boundaries to be very low (*P *= 7.81 × 10^-11^).

**Figure 3:**
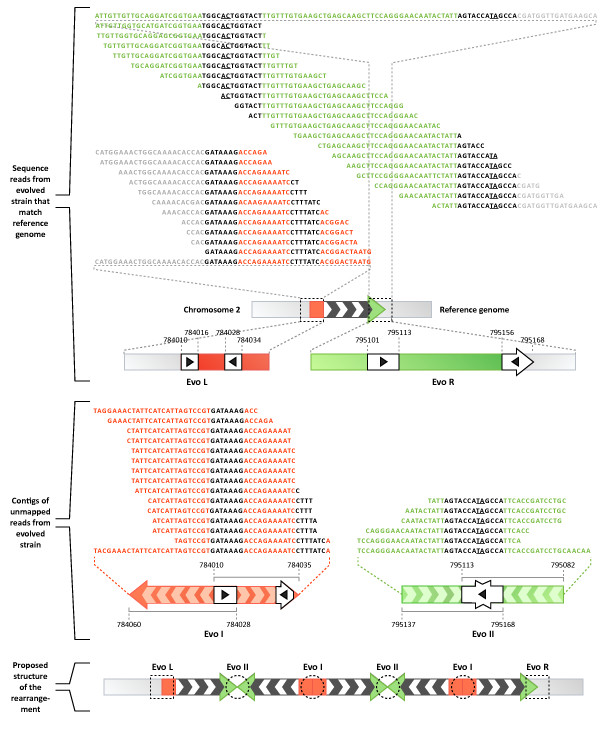
**Structural rearrangement and breakpoint information in the amplification of the *SUL1 *locus**. Top: Sequence reads from the evolved genome spanning the breakpoints of the chromosome 2 amplification (black and white arrow with red and green ends) are shown aligned to the wild-type genomic sequences at these termini. These reads support the presence of the wild-type sequences at the borders of the amplified segment: 'Evo L' (red) and 'Evo R' (green) ends. The positions of inverted repeats in the yeast genome are highlighted in white squares (with black arrow heads) with the corresponding coordinates from chromosome 2. Underlined bases in the right-end contigs and reads indicate positions where inverted repeats differ. Middle: Contigs of unmapped reads shown in red ('Evo I') and green ('Evo II') consist of chromosome 2 genomic sequences from the borders of amplification but contain an inversion breakpoint. Arrows indicate directionality of the subgenomic sequences composing these contigs. Black sequences correspond to the nearby inverted repeats in the reference genome. Coordinates of the regions of identity to the reference chromosome 2 are indicated below each contig. Left-end breakpoints ('Evo I') are composed of chr2: 784,010-784,035 and chr2: 784,028-784,060 subsequences inverted with a 7 nt overlap. Right-end breakpoints ('Evo II") are composed of chr2: 795,082-795,113 and chr2: 795,137-795,168 subsequences inverted with a 13 nt overlap. Bottom: Contig composition and the presence of reads spanning the wild-type sequences at the boundaries of the amplification support inverted rearrangements as the structure underlying the 5× amplification along chromosome 2.

The breakpoint-matching contigs are small (~50 bp) and contain inversions of nearby genomic sequences overlapped by ≤13 bp (Figure 3). These breakpoints occur within the *CTP1 *and *PCA1 *coding sequences, and would result in 192 and 797 amino acid truncations, respectively. In addition, we found reads spanning the wild-type sequence at these coordinates, indicating that full-length copies of these genes are retained in the genome of the adapted strain. In combination with the inversions in breakpoint-spanning contigs, the observation of reads that conform to the wild-type sequences across the predicted rearrangement boundaries suggests that the 5× amplification spanning chr2: 784,043-795,080 is structured as tandem inversions along the chromosome (Figure 3). This arrangement was validated by Southern blot analysis of the region with three different restriction enzymes and two probes [Additional File 2: Supplementary Figure S2]. The fact that at both breakpoints we found short homologous sequences 7-13 bp long overlapping the segmental inversions suggests that short homologous nucleotide tracts may be involved in driving the large structural rearrangements.

Among the contigs with subsequence alignments to the mappable reference genome, the preponderance were composed of mitochondrial sequences [Additional File 1: Supplementary Table S6]. This abundance of contigs of unmapped reads composed of mitochondrial subsequences was recapitulated with the ancestor genome data, but we found little overlap between structures observed in the two mitochondrial genomes [Additional File 1: Supplementary Table S7; Additional File 2: Supplementary Figure S3]. We did not observe copy number gains or losses at these coordinates, which are also covered at high-depth with wild-type sequences. These observations may reflect mitochondrial heteroplasmy in the form of structural rearrangements.

## Discussion

Evolutionary adaptation is a fundamental biological phenomenon with far-reaching implications in cancer, antibiotic resistance, infectious disease management, and ecology. Cataloging the precise genetic changes that underlie an organism's responses to defined selective environments can yield insights not only into the pathways that define fitness in these environments, but also into the breadth and reproducibility of adaptive mutations. The generation of such data requires approaches that can rapidly assay entire genomes with single-base resolution to expose point mutations and at megabase-scale to define structural rearrangements. In this study, we applied whole-genome sequencing to identify the genetic changes in a laboratory-evolved yeast strain, following on similar work in bacteria and phage [14,29-31]. This analysis revealed changes in the genome corresponding to single nucleotide polymorphisms and copy number amplifications, with specific gains over array-based methodologies.

We produced high-depth genome sequences for a yeast strain evolved under nutrient limitation and its parental strain. The genome sequence of the parent was critical in allowing us to pinpoint mutations acquired during experimental evolution, and serves as a template against which to compare the genomes of other descendent strains. We foresee the practice of maintaining high-quality genome sequences for stock strains in laboratories becoming pervasive as it facilitates rapid mutational profiling in laboratory evolution and selection experiments, and is readily applicable to a wide host of organisms and conditions.

We attempted a variety of filtering schemes for SNP-calling, incorporating thresholds for consensus base quality, mapping quality of supporting reads, and consensus quality of adjacent bases. However, these approaches generally reduced the coverage of the genome that could be analyzed or introduced false positives [Additional File 1: Supplementary Table S3]. We therefore adopted heuristics that allowed us to derive a set of high-confidence SNP calls while examining the majority of the haploid genome. This approach detected a novel and potentially regulatory SNP in the evolved genome near the *RRN3 *locus, encoding a nutrient-responsive transcriptional regulator, which is present at high frequency in the evolving population.

Read-depth information in high-throughput sequencing data yielded clear signals of copy number variation between strains. We applied a segmentation algorithm to refine the copy number estimate for the *SUL1 *amplification on chromosome 2. Following detection of copy number polymorphisms, we developed a general approach for detecting breakpoint sequences from single-end, short read sequencing data. Applied to the data of the genome of the evolved strain, this approach yielded upstream and downstream breakpoint predictions with single-base resolution for the *SUL1 *amplification. Using the derived breakpoint sequences, we predicted a ~55 kb genomic rearrangement supporting the copy number amplification of the *SUL1 *locus, and we validated this rearrangement experimentally [Additional File 2: Supplementary Figure S2]. The breakpoint sequences observed share the structural features of palindrome formation triggered by double-strand breaks near short inverted segments or by incorrect cleavage by a Holliday junction resolvase at an inverted repeat-mediated cruciform structure, and subsequent ligation of hairpins [32]. Narayanan et al. [33] have previously established this later mechanism in the development of inverted head-to-tail clusters of *CUP1 *and *SFA1 *between inserted Alu repeats in yeast centromeres. Thus, the observed breakpoint sequences in reads that match to two genomic locations provide insights into the structure of the segmental duplications and further evidence for the involvement of regions of micro-homology (≤15 bp) in driving large-scale genomic rearrangements [34].

## Conclusion

In this study, we demonstrate the ability of high-throughput sequencing to catalog the genetic changes underlying adaptation in a yeast strain evolved in a sulfate-limited environment. We generated high-depth genome sequences for evolved and parental yeast strains. We describe approaches effective at identifying single-nucleotide polymorphisms, as well as detecting the location of and quantifying copy number changes in the evolved genome with respect to that of its parental strain. Furthermore, we developed a general approach for detecting breakpoint sequences in single-end, short read sequencing data. This approach yielded breakpoint predictions with single base resolution for a ~11 kb amplification harboring the *SUL1 *gene, providing insights into the mechanisms that may have facilitated the expansion underlying this adaptive rearrangement. As such, this study suggests that the combination of whole-genome sequencing and experimental evolution is a powerful approach to study the features that restrain and promote genomic plasticity, defining possible routes of adaptation and outcomes of evolution. In addition, such studies should yield valuable functional information on the relationship between fitness and adaptations at both the single-gene and genomic levels [35]. Scaling these studies to population-level analysis will allow us to frame this knowledge within the context of population forces, which may yield new insights into evolutionary dynamics.

## Methods

DBY11331 (referred to as S2c1 in Gresham et al.) was isolated after ~188 generations of a sulfate-limited continuous culture seeded with the prototrophic haploid *S. cerevisiae *strain DBY10147 (*MATα*, *URA3*), as previously described [4]. Illumina sequencing libraries were constructed from DBY11331 and DBY10147 genomic DNA following standard procedures and published recommendations [36]. Briefly, 10 μg of yeast genomic DNA were sonicated to fragment sizes below 2000 bp, concentrated and end-repaired using the End-It DNA repair kit (EPICENTRE Biotechnologies). End-repaired DNA was A-tailed with GoTaq DNA polymerase (Promega) and ligated to Illumina adapters (QuickLigase, NEB). Ligation products between 300-400 bp were excised from a 6% polyacrylamide gel, eluted and ethanol precipitated. Fragment libraries were PCR amplified, cleaned following AMPure (Agencourt) and Qiaquick PCR clean-up procedures, and submitted for sequencing. We prepared two such libraries for each strain.

We collected 13,555,852 and 13,901,121 single-end, 36 bp, quality-filtered reads from DBY11331 and DBY10147, respectively, using the Illumina Genome Analyzer II platform. Reads were aligned to the UCSC sacCer1 reference sequence using *Maq *[23] with default parameters for single-end reads (12,274,183 evolved strain and 10,441,548 parental strain reads), to a coverage of ≥99.8%. We filtered reads with low mapping quality (score <10) and obtained a final coverage of ≥93.5% with an average read-depth of 35× and 28× in the non-gap regions of the evolved and parental genomes, respectively [Additional File 1: Supplementary Table S1].

For SNP-calling, we settled on a approach that required a nucleotide read depth ≥6× per position, with ≥80% base-calls supporting a SNP in the evolved genome data and ≥5× read depth, with ≥70% base-calls supporting a different base in the parental genome data. These nucleotide read depth thresholds allowed us to examine 90.99% of the mappable genome for SNPs [Additional File 1: Supplementary Table S2]. A parallel analysis relying on consensus base quality, quality of adjacent bases, and read mapping quality filters yielded similar SNP calls, but the fraction of the genome compliant with the analysis criteria was slightly reduced [Additional File 1: Supplementary Table S3].

We searched for small insertions and deletions by performing gapped alignment (*BLAT*) of the *Maq-*unmapped reads to the reference genome and recovering coordinates at which multiple unmapped reads show a bipartite alignment -an alignment to flanking sequences- as the best alignment. Candidate indel coordinates were reduced to sets specific to the evolved or ancestor genome. These strain-specific, candidate indels were then refined to maintain sites at which wild-type sequences are not observed in the *Maq*-alignment in the corresponding genome sequencing data, but are obtained in the comparison strain [Additional File 1: Supplementary Table S4].

To detect copy-number polymorphisms (CNPs), we averaged the per-nucleotide read depth data across 25 bp bins across the unique nuclear genome and normalized by the total nuclear bases acquired. For each bin, the log2-ratio in read depth between the evolved and parental data was calculated. Circular binary segmentation was applied on the ratios using *DNAcopy *[27], available as an R package, to partition the genome into regions of equal copy number. Segments were smoothed by removing changes < 3 standard deviations, and only those spanning ≥1000 bp were considered for further analysis. We used only one lane of data for each strain for copy number analysis (NCBI Sequence Read Archive accessions SRX014130 and SRX014132).

Breakpoint sequences for the *SUL1 *amplification were discovered as follows: Unmapped reads from the evolved genome data were assembled into contigs using *Velvet*, a *de novo *assembler for short-reads [28]. Contigs of unmapped reads were *BLAT*-aligned against the reference genome sequence requiring ≥90% un-gapped sequence identity. Alignments were filtered to remove contigs in which shared identity to a single genomic region spans the length of the contig, and contigs whose ends fall within the unmappable portions of the genome. In addition, we filtered contigs to remove those for which the subsequence alignments do not cover ≥90% of the contig. From this filtered group (11 contigs), seven contigs are composed of mitochondrial subsequences, three of nuclear subsequences, and one shares sequence identity to nuclear and mitochondrial sequences [Additional File 1: Supplementary Table S6]. The two contigs aligned to amplification boundary coordinates were selected as candidate breakpoint sequences for the *SUL1 *amplification and examined in detail. We estimated the probability of these candidate breakpoint contigs arising independently of the amplification by analyzing contigs of unmapped reads derived from the ancestor genome data. Briefly, contigs of ancestor genome unmapped reads were assembled and aligned to the reference genome sequence. This yielded two contigs of unmapped reads composed of sequences with alignments to the mappable nuclear genome coordinates [Additional File 1: Supplementary Table S7]. The probability of observing such contigs was then calculated per mappable base in the nuclear genome. We limit this estimate to the nuclear genome to account for differences in the read-depth between the nuclear and mitochondrial genomes.

For Southern blot analysis, genomic DNA was digested overnight with BamHI, EcoRV or PstI (New England Biolabs). Samples were subjected to electrophoresis through 0.6% w/v agarose in 1× TBE overnight at 33 V, visualized after ethidium bromide staining and transferred to a GeneScreen™ hybridization transfer membrane (PerkinEilmer) in 10× SSC. Hybridization was performed at 65°C for approximately 20 h with ^32^P-labeled "SUL1" and "BamHI" probes constructed by PCR [Additional File 1: Supplementary Table S5].

Tiling array SNP analysis and ORF array CGH data were obtained as previously described [4].

### Data deposition

Data are archived at NCBI Sequence Read Archive (SRA) under accession SRP001478.

## Abbreviations

(SNP): Single-nucleotide polymorphism; (CNP): copy-number polymorphism; (CNV): copy-number variation; (CGH): comparative genome hybridization.

## Authors' contributions

CA, MD and SF conceived of and designed the experiments and wrote the manuscript. CA performed all sequencing experiments and computational analysis. CP performed Southern blot experiments. All authors read and approved the final manuscript.

## Supplementary Material

Additional file 1 **Supplementary Tables**. This file contains the supplementary tables with information on sequencing and mapping statistics, SNP calling, small insertion and deletion screening, Southern blot probe specifications, and alignment information for contigs of unmapped reads. Click here for file

Additional file 2 **Supplementary Figures**. This file contains supplementary figures including a per-chromosome view of the segmentation analysis, Southern blot experimental results, and views of alignments for contigs of unmapped reads. Click here for file
