# Supplementary material for: Whole-genome sequencing of a laboratory-evolved yeast strain
Source: BMC Genomics. 2010 Feb 3;11:88. doi: 10.1186/1471-2164-11-88 (PMC2829512; doi:10.1186/1471-2164-11-88)
Supplement: Additional file 1 — Supplementary Tables. This file contains the supplementary tables with information on sequencing and mapping statistics, SNP calling, small insertion and deletion screening, Southern blot probe specifications, and alignment information for contigs of unmapped reads. [file 1471-2164-11-88-S1.PDF]

**Supplementary Table S1. Sequencing data acquisition and mapping statistics.**

| | | Evolved | Parent |
|---|---|---|---|
| Data acquistion | Library #1 reads: | 6,070,144 | 6,664,264 |
| | Library #2 reads: | 7,485,708 | 7,236,857 |
| | Combined reads: | 13,555,852 | 13,901,121 |
| Mapping statistics | Yeast genomic reads: | 12,274,183 | 10,441,548 |
| | QC mapped reads: | 10,123,706 | 8,818,796 |
| | Nuclear genome coverage: | 93.55% | 93.51% |
| | Mappable nuclear genome coverage: | 99.82% | 99.78% |
| | Depth in nuclear non-gaps: | 35.0x | 28.23x |
| | Mitochondrial genome coverage: | 97.01% | 97.02% |
| | Mappable mitochondrial genome coverage: | 99.99% | 99.99% |
| | Depth in mitochondrial non-gaps: | 531.59x | 666.34x |

**Supplementary Table S2. Summary of SNP calling results obtained with filters for read-depth and base call frequency.**

| Read-depth threshold | | | Frequency of concordant base calls | | | Results | | | |
|---|---|---|---|---|---|---|---|---|---|
| **Evolved genome** | **Ancestor genome** | **Compliant bases (%)** | **Evolved genome** | **Ancestor genome** | **Compliant bases (%)** | **Analyzed bases (%)** | **SNP calls** | **False positives** | **False negatives** |
| 3 | 3 | 92.0 | ≥0.8 | ≥0.7 | 94.0 | 91.8 | 13 | + | – |
| 4 | 4 | 89.8 | ≥0.8 | ≥0.7 | 94.0 | 89.7 | 8 | + | – |
| 5 | 6 | 86.9 | ≥0.8 | ≥0.7 | 94.0 | 86.8 | 5 | + | – |
| 6 | 6 | 83.5 | ≥0.8 | ≥0.7 | 94.0 | 83.4 | 4 | – | – |
| | | | | | | | | | |
| 6 | 5 | 85.4 | ≥0.8 | ≥0.7 | 94.0 | 85.3 | 4 | – | – |
| 7 | 5 | 83.5 | ≥0.8 | ≥0.7 | 94.0 | 83.4 | 4 | – | – |
| 8 | 5 | 81.2 | ≥0.8 | ≥0.7 | 94.0 | 81.1 | 4 | – | – |

The number of bases compliant in both genomes for each filter is indicated, as is the coverage of the analysis across the genome. Number of SNP calls, and presence (+) and absence (-) of false positives/negatives is indicated. (*) Indicates the selected parameters for final analysis.

**Supplementary Table S3. Summary of selected SNP calling results obtained with varying thresholds for commonly used parameters.**

| Read depth | | Base consensus quality | | Adjacent base qualities | | Read mapping quality | | Results | | | |
|---|---|---|---|---|---|---|---|---|---|---|---|
| **Threshold** | **Compliant bases (%)** | **Threshold** | **Compliant bases (%)** | **Threshold** | **Compliant bases (%)** | **Threshold** | **Compliant bases (%)** | **Analyzed bases (%)** | **SNP calls** | **False positives** | **False negatives** |
| 3 | 92.0 | 90 | 90.0 | 30 | 93.8 | 30 | 94.0 | 89.9 | 11 | + | – |
| 3 | 92.0 | 100 | 88.9 | 30 | 93.8 | 30 | 94.0 | 88.7 | 9 | + | – |
| 3 | 92.0 | 110 | 87.6 | 30 | 93.8 | 30 | 94.0 | 87.5 | 5 | + | – |
| 3 | 92.0 | 120 | 86.2 | 30 | 93.8 | 30 | 94.0 | 86.1 | 5 | + | – |
| 3 | 92.0 | 130 | 84.5 | 30 | 93.8 | 30 | 94.0 | 84.5 | 4 | – | – |
| 3 | 92.0 | 140 | 82.8 | 30 | 93.8 | 30 | 94.0 | 82.7 | 3 | – | + |
| 3 | 92.0 | 150 | 80.8 | 30 | 93.8 | 30 | 94.0 | 80.7 | 3 | – | + |
| 4 | 89.8 | 90 | 90.0 | 30 | 93.8 | 30 | 94.0 | 89.0 | 11 | + | – |
| 4 | 89.8 | 100 | 88.9 | 30 | 93.8 | 30 | 94.0 | 88.4 | 9 | + | – |
| 4 | 89.8 | 110 | 87.6 | 30 | 93.8 | 30 | 94.0 | 87.4 | 5 | + | – |
| 4 | 89.8 | 120 | 86.2 | 30 | 93.8 | 30 | 94.0 | 86.1 | 5 | + | – |
| 4 | 89.8 | 130 | 84.5 | 30 | 93.8 | 30 | 94.0 | 84.5 | 4 | – | – |
| 4 | 89.8 | 140 | 82.8 | 30 | 93.8 | 30 | 94.0 | 82.7 | 3 | – | + |
| 4 | 89.8 | 150 | 80.8 | 30 | 93.8 | 30 | 94.0 | 80.7 | 3 | – | + |
| 5 | 86.9 | 90 | 90.0 | 30 | 93.8 | 30 | 94.0 | 86.5 | 11 | + | – |
| 5 | 86.9 | 100 | 88.9 | 30 | 93.8 | 30 | 94.0 | 86.3 | 9 | + | – |
| 5 | 86.9 | 110 | 87.6 | 30 | 93.8 | 30 | 94.0 | 86.0 | 5 | + | – |
| 5 | 86.9 | 120 | 86.2 | 30 | 93.8 | 30 | 94.0 | 85.3 | 5 | + | – |
| 5 | 86.9 | 130 | 84.5 | 30 | 93.8 | 30 | 94.0 | 84.2 | 4 | – | – |
| 5 | 86.9 | 140 | 82.8 | 30 | 93.8 | 30 | 94.0 | 82.6 | 3 | – | + |
| 5 | 86.9 | 150 | 80.8 | 30 | 93.8 | 30 | 94.0 | 80.7 | 3 | – | + |

Filters were applied to the each position in the ancestor and evolved genome data. These include: minimum read-depth cutoff, and thresholds for the *Phred*-scaled probability scores of consensus base call at position and adjacent +/-3 bases, as well as for the alignment of reads covering position. The fractions of compliant bases are shown for each threshold individually and combined ('Analyzed bases'). The number of SNP calls and presence of false positives/negatives is indicated for each setting.

**Supplementary Table S4. Summary of small insertion and deletions detected in *BLAT* alignments of unmapped reads.**

| Strain | Chrom. | Position | *BLAT* reads | Size (bp) | Type | Mapping positions | Context | *Maq* reads | Span | WT | Span | WT |
|---|---|---|---|---|---|---|---|---|---|---|---|---|
| | | | | | | | | (found in self) | | | (comparison) | |
| Evolved | chr7 | 485899 | 3 | 1 | Insert | 3 | Intergenic | 1 | 0 | 0 | 3 | 2[A] |
| Parent | chr4 | 807790 | 3 | 1 | Deletion | 2 | Intergenic | 0[B] | 0[B] | 0[B] | 8 | 8 |

Candidate indels were first selected requiring a minimum of 3 supporting ungapped alignments with concording coordinates, with at least 2 distinct mapping coordinates. We refined strain-specific candidate indel coordinates to the subset for which *Maq*-aligned reads with quality score ≥30 do not support wildtype sequences in the strain but do support wildtype sequences in the comparison strain. We define a read as indel-spanning if its alignment covers the indel coordinate by 4bp on both sides. The number of such spanning reads for each coordinate is indicated (Span). Coordinates are defined as wildtype (WT) if at least one quarter of the indel-spanning reads contain the UCSC sacCer1 reference genome sequence spanning the indel +/- 4bp. [A] Two of the spanning reads support an indel at this site in the parent genome. [B] A read with quality score <30 from the parental genome sequencing data is wildtype for these coordinates, suggesting this position is in fact wildtype for this strain.

**Supplementary Table S5. Primer sequences for PCR construction of Southern blot probes.**

| Primer | Primer sequence | Probe | Target coordinates | Product size (bp) |
|---|---|---|---|---|
| **Probe1-for:** | GCTGATTTAGTGGCAGGAATT | “SUL1” | chr2:789,554-790,447 | 894 |
| **Probe1-rev:** | GTAGTCGTTAATTCTACCAAAG | “SUL1” | | |
| **Probe2-for:** | ATTATGGTAAAGTGTGGTGA | “BamHI” | chr2:785,960-786,557 | 598 |
| **Probe2-rev:** | CTGTGGATTTGCACACAATC | “BamHI” | | |

**Supplementary Table S6. Alignment features for filtered contigs assembled from evolved genome unmapped reads .**

| Contig ID | Alignment coordinates | Strand | Contig size (nt) | Match size (nt) | Start | End | Mis-match |
|---|---|---|---|---|---|---|---|
| NODE_11509_length_39_cov_4.692308 | chrM:39930-39966 | - | 55 | 34 | 2 | 38 | 2 |
| | chr6:229889-229918 | + | 55 | 29 | 26 | 55 | 0 |
| NODE_12204_length_77_cov_7.701299 | chr2:636246-636300 | + | 93 | 49 | 0 | 54 | 5 |
| | chr11:255380-255426 | - | 93 | 46 | 46 | 93 | 0 |
| NODE_5703_length_34_cov_6.470588 | chrM:26311-26339 | + | 50 | 28 | 22 | 50 | 0 |
| | chrM:26345-26375 | - | 50 | 30 | 0 | 30 | 0 |
| NODE_3479_length_78_cov_5.076923 | chrM:26558-26619 | - | 94 | 61 | 33 | 94 | 0 |
| | chrM:19141-19192 | + | 94 | 49 | 0 | 50 | 1 |
| NODE_3608_length_54_cov_6.629630 | chrM:21273-21322 | - | 70 | 48 | 21 | 70 | 1 |
| | chrM:21377-21407 | + | 70 | 30 | 0 | 30 | 0 |
| NODE_9229_length_36_cov_15.888889 | chr2:784009-784035 | - | 52 | 26 | 0 | 26 | 0 |
| | chr2:784027-784060 | + | 52 | 33 | 19 | 52 | 0 |
| NODE_14235_length_35_cov_4.942857 | chr2:795136-795168 | - | 51 | 32 | 19 | 51 | 0 |
| | chr2:795081-795113 | + | 51 | 30 | 0 | 32 | 2 |
| NODE_50_length_97_cov_10.113402 | chrM:79796-79862 | + | 113 | 66 | 0 | 66 | 0 |
| | chrM:28925-29129 | + | 113 | 76 | 17 | 113 | 1 |
| NODE_1751_length_69_cov_23.086957 | chrM:17071-17128 | - | 85 | 49 | 36 | 85 | 0 |
| | chrM:17080-17132 | + | 85 | 48 | 0 | 52 | 4 |
| NODE_1177_length_35_cov_5.485714 | chrM:36712-36747 | + | 51 | 34 | 0 | 37 | 0 |
| | chrM:36727-36754 | - | 51 | 27 | 24 | 51 | 0 |
| NODE_11789_length_96_cov_5.312500 | chrM:14890-14931 | + | 112 | 41 | 0 | 41 | 0 |
| | chrM:14841-14904 | - | 112 | 63 | 38 | 101 | 0 |

**Supplementary Table S7. Alignment features for filtered contigs assembled from ancestor genome unmapped reads.**

| Contig ID | Alignment coordinates | Strand | Contig size (nt) | Match size (nt) | Start | End | Mis-match |
|---|---|---|---|---|---|---|---|
| NODE_11727_length_87_cov_8.908046 | chrM:22225-22281 | + | 103 | 56 | 47 | 103 | 0 |
| | chrM:22082-22139 | - | 103 | 56 | 0 | 57 | 1 |
| NODE_3506_length_48_cov_19.854167 | chr6:93685-93722 | + | 64 | 35 | 0 | 36 | 0 |
| | chr10:460183-460223 | + | 64 | 39 | 25 | 64 | 0 |
| NODE_11211_length_75_cov_9.693333 | chrM:41363-41408 | + | 91 | 45 | 46 | 91 | 0 |
| | chrM:41399-41449 | - | 91 | 49 | 6 | 56 | 1 |
| NODE_4121_length_38_cov_4.263158 | chrM:6890-6924 | + | 54 | 34 | 0 | 35 | 0 |
| | chrM:6914-6942 | - | 54 | 28 | 26 | 54 | 0 |
| NODE_12617_length_68_cov_11.250000 | chr2:631933-631972 | + | 84 | 37 | 44 | 84 | 0 |
| | chr2:631859-631916 | - | 84 | 52 | 0 | 57 | 5 |
| NODE_8710_length_58_cov_10.931034 | chrM:26311-26353 | - | 74 | 42 | 32 | 74 | 0 |
| | chr7:404946-404976 | + | 74 | 30 | 32 | 62 | 0 |
| | chrM:26286-26319 | + | 74 | 33 | 6 | 40 | 0 |
| NODE_3266_length_43_cov_15.953488 | chrM:60638-60683 | - | 59 | 40 | 9 | 59 | 0 |
| | chrM:59016-59052 | - | 59 | 36 | 0 | 36 | 0 |
| NODE_1804_length_61_cov_13.557377 | chrM:79695-79727 | + | 77 | 31 | 0 | 32 | 1 |
| | chrM:46903-46958 | + | 77 | 55 | 22 | 77 | 0 |
