# Supplementary material for: Whole-genome sequencing of a laboratory-evolved yeast strain
Source: BMC Genomics. 2010 Feb 3;11:88. doi: 10.1186/1471-2164-11-88 (PMC2829512; doi:10.1186/1471-2164-11-88)
Supplement: Additional file 2 — Supplementary Figures. This file contains supplementary figures including a per-chromosome view of the segmentation analysis, Southern blot experimental results, and views of alignments for contigs of unmapped reads. [file 1471-2164-11-88-S2.PDF]

Supplementary Figure Legends

Supplementary Figure S1. Segmentation of chromosomes based on read-depth ratios between evolved and ancestor genomes. Segmentation analysis of read-depth data between evolved and ancestor genomes is presented for each chromosome. Black squares correspond to the $\log_2$ ratio of the evolved/ancestor read-depth across 25 nt windows of mappable bases bases in the genome. The red line corresponds to the circular binary segmentation-derived regions of equal copy number. Segments are smoothed by removing splits corresponding to changes < 3 standard deviations. As shown, unmappable coordinates are not accounted for in the segmentation. This analysis was performed using data only from one library from each strain (NCBI Sequence Read Archive accessions SRX014130 and SRX014132).

Supplementary Figure S2. Experimental validation of the proposed structural rearrangement. A) Southern blot gels for hybridizations with probes “SUL1” and “BamHI” of PstI, EcoRV, and BamHI digested genomic DNA from parental (P) and evolved (E) strains. Fragment sizes in base pairs are indicated. B) Expected sizes (in base pairs) and number (in parenthesis) of probe-bearing fragments from evolved and parental genomic DNA after digestion with PstI, EcoRV and BamHI.

Supplementary Figure S3. Alignment of contigs of unmapped reads to the mitochondrial genome. Top left: Chromosome-level view of alignments from contigs of unmapped reads from the evolved genome to the mitochondrial genome. Alignments to the plus strand are displayed as green arrows, whereas red arrows correspond to minus strand alignments. Gray dots correspond to the $\log_2$ ratio of the read-depth between the evolved and parental genome data sets averaged in 5 bp bins. The segmentation predicted copy number ratio between genomes is shown (red line). Top right:

Chromosomal view of the alignments of contigs of unmapped ancestor genome reads to the mitochondrial genome. As before, plus strand and minus strand alignments are indicated by green arrows and red arrows, respectively. Read-depth ratio and segmentation defined regions of equal copy number are shown in gray dots and red lines, respectively. Middle and bottom left: Close-up view of the alignment coordinates for example contigs of unmapped reads from the evolved genome data. Green and orange correspond to the read-depth per hundred-thousand chromosomal bases acquired for the evolved and ancestor genome data, respectively. Middle and bottom right: Close-up view of the alignment coordinates for sample contigs of unmapped ancestor genome reads. As above, green and orange correspond to the read-depth per hundred-thousand chromosomal bases acquired for the evolved and ancestor genome data, respectively.

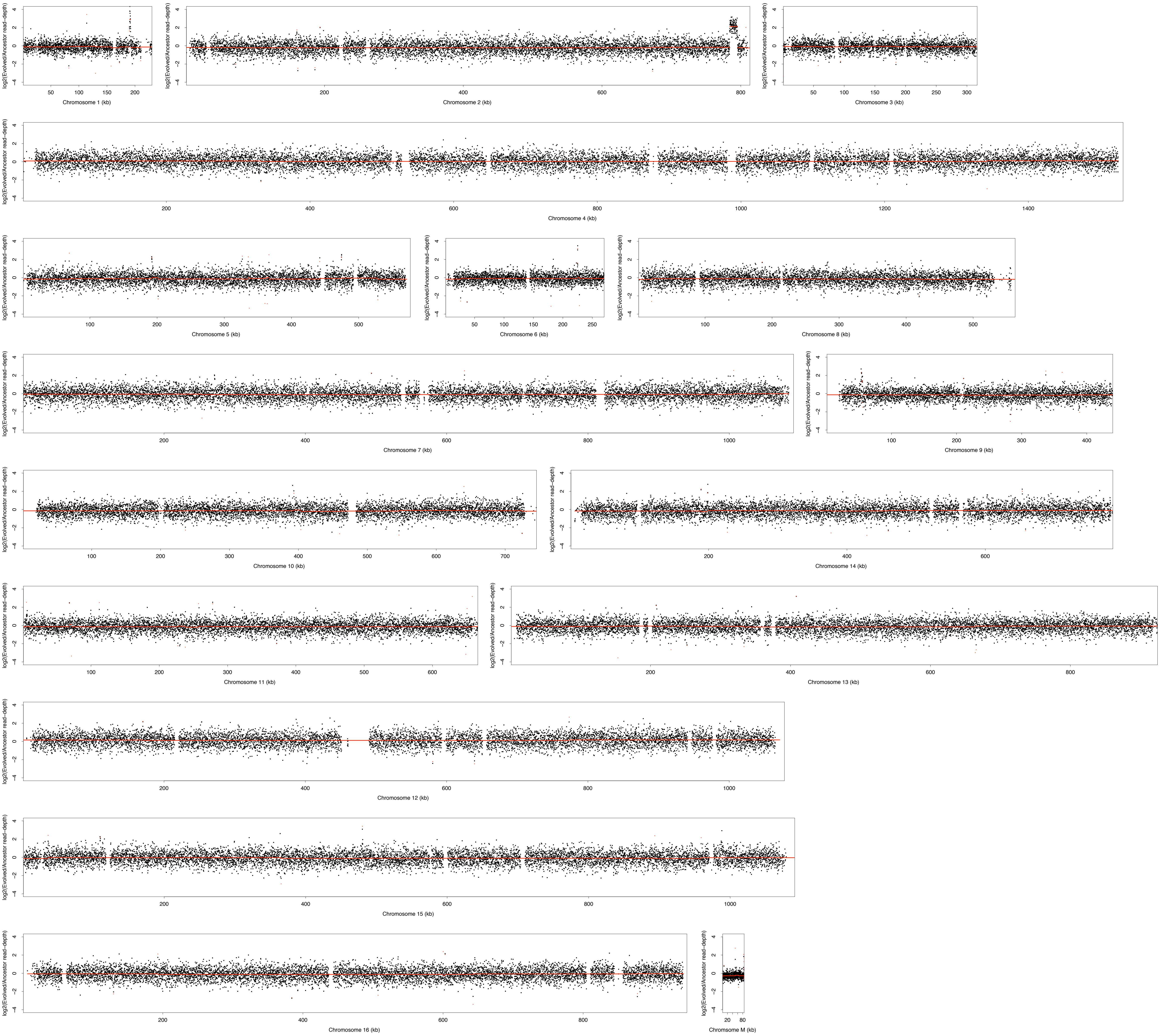

Supplementary Figure S1

A

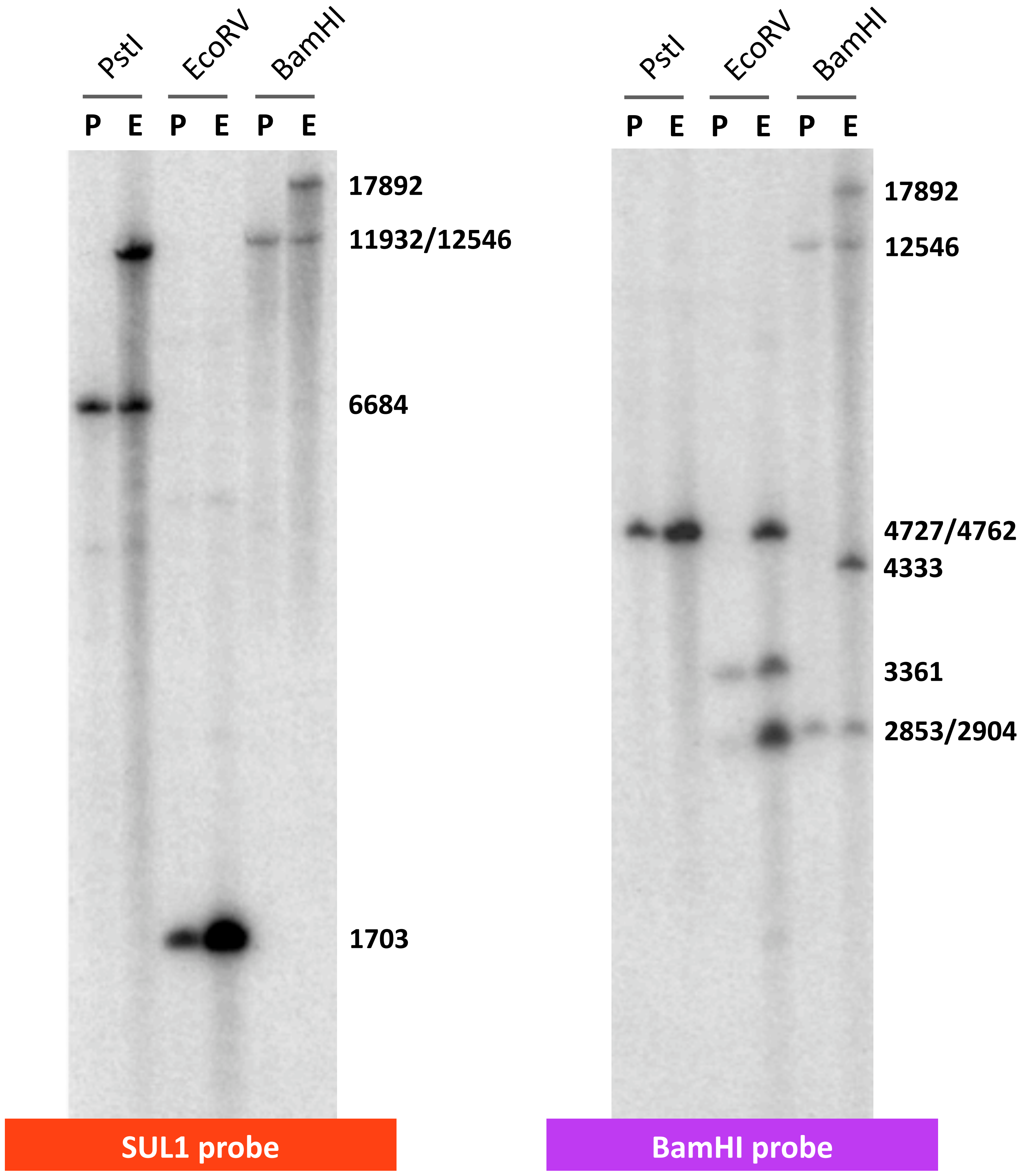

B

	Parent (P)	Evolved (E)	Parent (P)	Evolved (E)
**PstI:**	6684	11932(2), 6684	4762	4762(5)
**EcoRV:**	1703	1703(5)	2853, 3361	4727(2), 3361, 2853(5)
**BamHI:**	12546	17892(2), 12546	2904, 12546	2904, 4333(2), 12546, 17892(2)

Supplementary Figure S2

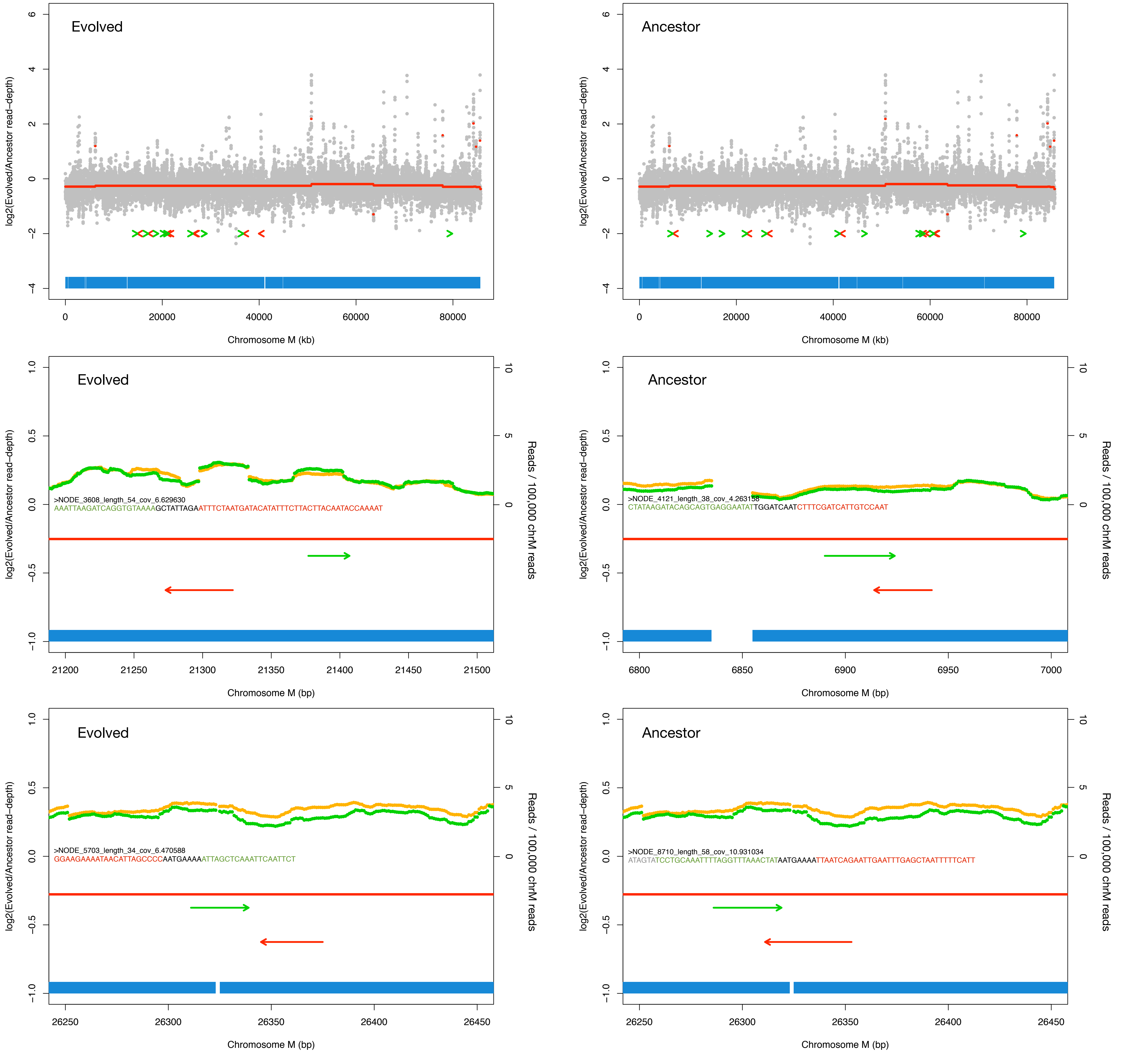

Supplementary Figure S3
